# Authors' Responses to Peer Reviews of “Supporting Technologies for COVID-19 Prevention: Systemized Review”

**DOI:** 10.2196/38693

**Published:** 2022-05-24

**Authors:** Zhuo Zhao, Rui Li, Yangmyung Ma, Iman Islam, Abdul M Azam Rajper, WenZhan Song, Hongliang Ren, Zion Tsz Ho Tse

**Affiliations:** 1 School of Electrical and Computer Engineering University of Georgia Athens, GA United States; 2 Tandon School of Engineering New York University Brooklyn, NY United States; 3 Hull York Medical School University of York Heslington York United Kingdom; 4 Department of Computer Science University of Georgia Athens, GA United States; 5 Department of Biomedical Engineering National University of Singapore Singapore Singapore; 6 Department of Electronic Engineering University of York York United Kingdom

**Keywords:** COVID-19, medical treatments, personal protective equipment, testing methods

*This is the authors’ response to peer-review reports for "Supporting Technologies for COVID-19 Prevention: Systemized Review".*

## Round 1 Review

### Anonymous [1]

#### General Comments

The manuscript [2] talks about medical technologies during COVID-19. The review is nice to read. I could not find Table 2.

#### Specific Comments

##### Major Comments

1. My main concern is that several technologies are missing, so I am not sure if the review on Google search was carried out properly. There must definitely be over 90 technologies. If you check the Federal Drug Administration (FDA) In Vitro Diagnostics, there are over 240 test kits alone. Additionally, I am not sure how you reach to 38 items from 90, or are there so many unrelated items?

Authors' response: The title has been adjusted to narrow down the search range. Moreover, detailed selection criteria have been included.

2. The images in the figures, especially on company products, need actual permission from the original company or inventor. For example, the image citing reference 2 is a British Broadcasting Corporation (BBC) article, but the actual image is from a hospital whose permission is needed, rather than citing BBC.

Authors' response: Proper citation has been done through the company website.

3. Several topics are outdated as of now, such as personal protective equipment. The interest in smart or green personal protective equipment has declined dramatically as vaccination has picked up. Therefore, the text needs to be made aligned to current needs, such as low-temperature storage technologies to store vaccines, etc.The ventilators section is interesting, but such images have been shown before in many places. As such, it will be difficult to garner readership based on the sections.

Authors' response: The vaccine storage is very interesting. However, due to the length of this review paper, it is difficult to explore a whole new different topic.

4. Several points are repeated throughout the manuscript, such as lack of manpower and resources. The flow of the text could be made more fast paced by removing general statements and sticking to facts only.

Authors' response: The manuscript has been checked to avoid general statements.

5. New and interesting topics could be added based on the current status of the pandemic, such as technologies centering around vaccination or at-home testing.

Authors' response: At-home testing has been mentioned in the article.

### Reviewer CM [3]

#### General Comments

The need for effective and rapid response mechanisms to the COVID-19 pandemic has seen the emergence of new technologies. The European Parliament has organized such technologies into 10 broad categories. Many studies have reported the emergence of new digital tools as a direct response to COVID-19. While some of the studies report that these technologies make a major impact in the management of COVID-19 despite some challenges in their real-life usage, others acknowledge that COVID-19 control is critical, which calls for regular stock-taking, given the rapid advances in the field. Following the above, the authors of the paper “Supporting Technologies for COVID-19 Prevention: Systemized Review,” [2] in an attempt to stay on top of these advances, investigated the emerging technologies relating to the COVID-19 pandemic. The topic addressed in this paper is of interest to the journal’s readership and the international community. Being an important topic, it would have been important to report the review based on specific reporting guidelines to make it more appealing. The paper does not comply with the journal guidelines. Apart from the lack of a research objective, the paper is lacking in its methodology due to the lack of use of reporting guidelines. As such, the results remain doubtful. The general structure and English warrant improvement. If this paper must be brought to standard, the following specific comments are worth considering;

#### Specific Comments

1. The title of the paper does not conform to the journal guidelines.

Authors' response: The title has been adjusted.

2. The Abstract of your paper needs to be structured following the recommended guidelines.

Authors' response: The abstract has been adjusted.

3. This paper neither has a research objective nor question to permit its evaluation.

Authors' response: research objective has been added.

4. You need to follow the guidelines of the journal to which you are submitting.

Authors' response: The guideline has been double-checked.

5. Kindly refer to the new PRISMA checklist to see how you can report your search results.

Authors' response: The PRISMA checklist has been double-checked.

6. You need to have a look at the reviews published in the journal you are submitting to.

Authors' response: The newly published work has been double-checked.

7. The English of your paper needs to be improved.

Authors’ response: English has been checked thoroughly.

8. The Methods section lacks clarity and warrants improvement.

Authors’ response: The method section has been improved.

9. Your references need to be in line with the journal guidelines.

Authors’ response: The references have been edited.

The above specific comments are further divided into the below major and minor comments;

##### Major Comments

1. Firstly, you need to identify and report the type of review you conducted to help in the evaluation of your paper. If this is a narrative review, kindly indicate clearly in your paper

2. I suggest the following: (1) Emerging Medical Technologies for Fighting COVID-19: Systematic Review; or (2) Emerging Medical Technologies for Fighting COVID-19: Narrative Review

3. Your abstract needs to be structured in line with the journal guidelines, to include the Background, Objective, Methods, Results, and Conclusion subsections. Additionally, be aware that the PRISMA checklist also provides additional information that must appear in the Abstract section of systematic reviews.

Authors’ response: The title has been changed to: Systematic Review of Supporting Technologies for COVID Prevention

3. Your abstract needs to be structured in line with the journal guidelines, to include the Background, Objective, Methods, Results, and Conclusion subsections. Additionally, be aware that the PRISMA checklist also provides additional information that must appear in the Abstract section of systematic reviews.

Authors’ response: The abstract has been restructured.

4. Kindly restructure the manuscript using the IMRD format using the following word template;

Authors’ response: The journal guideline has been checked.

5. It is absolutely important to read through the journal guidelines to which you are submitting.

Authors’ response: The journal guideline has been checked.

6. Kindly put your study in context as part of your introduction. Use the provided reference if you need help with how to put your study in context.

Authors’ response: The reference has been used to add more content to the introduction.

7. This study is without a research objective. State your research question and objectives.

Authors’ response: The research objective has been added to the abstract.

8. Kindly report the Methods section using the subsections below:

- Study objectives
- Eligibility criteria for selected studies
- How literature was searched
- The method used to synthesize results
- Data management and analysis
- Quality assessment (including the risk of bias assessments)
- How missing data were handled
- Heterogeneity assessment
- The method used to present data and results

The above may vary depending on the type of review you undertook. A simple literature review of emerging technologies will normally not require some of the above subsections.

Authors’ response: The Methods section has been restructured to include some of the bullet points above.

9. It is very important to indicate the guidelines used to report your review results.

Authors’ response: The guideline PRISMA 2020 has been mentioned in the Methods section.

10. Your results section should be reported based on your research objectives (yet to be defined), and should include the following:

a. Search results: [a] flow diagram based on the new PRISMA flow chart and [b] characteristics of included studies (table and discussion).

b. Risk of bias assessment

c. Synthesis results (report results based on objectives and the different technology categories)

d. Overall assessment of the body of evidence

e. Heterogeneity

Again, as highlighted above, a literature review will not require some of the above points (assessment of overall evidence, and heterogeneity). That said, if you did a narrative review, I suggest using the following reported guidelines. I also find the structure of this referenced narrative review and systematic review more robust (use these as references in reporting your review). In reporting a narrative review, it is important to bear in mind how narrative reviews are evaluated. Moreover, be aware that review papers are expected to be submitted with a filled template of the guidelines used.

Authors’ response: The research objectives have been defined in the Methods section. The new PRISMA flow chart has been added into the manuscript.

11. You need to have a look at studies that have reported on similar topics for inspiration.

Authors’ response: The articles have been thoroughly read and cited in the manuscript.

12. See guidelines for the structure of the Discussion section. Present your Discussion into (1) Principal findings and (2) Comparison with prior studies.

Authors’ response: The Discussion section has been reorganized.

13. Kindly include a subsection “study limitations” as part of the Discussion section.

Authors’ response: The study limitation has been included as part of the Discussion section.

14. Your references have to be in line with the recommended journal guidelines. Set your reference manager to the AMA citation style and make sure to include a PubMed ID at the end of each reference. You can search the PubMed IDs of various articles at https://pubmed.ncbi.nlm.nih.gov/. In the absence of a PubMed ID, kindly include a DOI (verify your DOIs using https://www.doi.org/).

Authors’ response: The reference has been checked.

15. Include a subsection “Author Contribution” after the Acknowledgments section to state the contribution of each author included in this paper.

Authors’ response: The section has been added.

16. Include a subsection “Conflicts of Interest” after Author contributions to declare any conflict of interest.

Authors’ response: The section has been added.

17. Kindly list all Multimedia Appendices before the References.

Authors’ response: No Multimedia Appendices in this manuscript.

18. For referenced websites, ensure to make as much effort as possible to get and reference the pdf version of the article (ie, in the absence of a PMID and DOI).

Authors’ response: PDF of the websites have been added.

19. Create a section “Abbreviations” after your references to list and expand all abbreviations in the text.

Authors’ response: The section “Abbreviations” has been added.

20. I suggest starting your Conclusion with a statement on the study objectives followed by a summary of findings, then lessons learned from your findings, and finally, suggested direction of future research.

Authors’ response: The conclusion has been restructured.

##### Minor Comments

1. Kindly include only the corresponding author in the manuscript and create or include all coauthors in the metadata section of the online manuscript management system (MMS) of your journal profile.

Authors’ response: The title page has been changed accordingly.

2. End your introduction with the aim of the study.

Authors’ response: The aim of the study has been added.

3. Kindly format your table following the journal guidelines.

Authors’ response: The table has been reformatted according to the guidelines.

4. You may want to start your Table 1 with study id, by merging columns 1, 2, and the last as 1 column. For instance, the first cell will be Rendeki et al (2020), followed by the “setting or country” in the second column, and then the description, etc.

Authors’ response: Table 1 has been restructured.

5. Following from (4) above, I recommend having (1) a table of characteristics of included studies for each category of technology or (2) present a single table of “Characteristics of included studies” under the Search Results subsection of the Results section, after the PRISMA flow diagram.

Authors’ response: The table of characteristics has been added for each category of technology.

6. I suggest attempting to format your Figure 1 following the new PRISMA diagram.

Authors’ response: Figure 1 has been reformatted.

7. Review all your figures and their captions in line with the guidelines. Apart from being uploaded as Multimedia Appendices, all figures must appear in the body of the text where they are first mentioned. Use a single sentence as the caption for each figure, which should appear at the bottom of the figure.

Authors’ response: The figures have been placed next to the paragraph where they were first mentioned. The figure caption has been checked and edited.

8. Following from (7) above, you may want to combine figures (a) to (i) to form a single figure as is the case with Figure 4.

Authors’ response: Figure 2-3 has been checked and ensured consistency with Figure 4.

9. I advise downloading Grammarly to assist you with the editing of your paper.

Authors’ response: Grammarly has been used.

10. There is a need to justify your outcome prioritization. I suggest organizing your technology categories in line with the European Parliament categorization.

Authors’ response: 3D printed facial mask has been prioritized in the manuscript.

11. Ensure that titles and subtitles of your “Comparison with Prior studies” subsection of the Discussion are the same as the titles and subtitles of your Results section (Prevention, Diagnosis, Treatment, etc), and as suggested in (30) above.

Authors’ response: The discussion section has been reorganized.

## Round 2 Review

We would like to express our gratitude once again to the reviewer for detailed thoughts and feedback. We have carefully considered, responded to, and made changes to the manuscript based on the specific recommendations of the reviewers. We feel that the changes have greatly increased the overall quality of work, and we are very appreciative of the kind comments. Thank you. Our responses to the specific comments of the reviewers are listed as follows.

### Reviewer CM [3]

#### General Comments

I acknowledge that the authors of the paper titled “Supporting Technologies for COVID-19 Prevention: Systemized Review”[2] have done well to improve on the overall structure and presentation of the paper, with a much better flow. Comments that were made in the previous round were based on the understanding that this was a standard “systematic review” type paper, but this is not the case. However, this paper still warrants some improvement. Kindly refer to the below major comments.

#### Specific Comments

##### Major Comments

1. Systematic reviews require a predefined robust search strategy that is exhaustive, an appraisal scheme for each type of study (both risk of bias and quality) with well-cited tools, a clearly outlined method for synthesizing results, a method of assessing all the evidence emanating from the literature and most especially, with a clearly stated guideline used in reporting the review. Given that this review does not formally appraise the included studies for risk of bias and quality, neither does it have a clearly outlined method of synthesis, It will be appropriate to identify your study either as a (1) literature review, (2) systemized review, (3) narrative review, or simply (4) overview, all of which do not forcefully require a comprehensive search, formal appraisal of studies and typically aimed at a narrative synthesis [1]. It is enough to note here that even “systematic reviews with narrative synthesis” and “rapid reviews” that may omit some aspects of a standard systematic review, follow specific citable guidelines in their methods and synthesis approach, to say the least.

Authors' response: The paper title has been changed to “Supporting Technologies for COVID-19 Prevention: Systemized Review.”

2. It is absolutely important to bear in mind that reviews have their terminologies, as is the case with randomized controlled trials or other studies. You wrote I quote “In this paper, 150 news articles and scientific reports on COVID-19-related innovations during 2020-2021 were firstly checked, screened and shortlisted to form a pool of candidates yielding a total of 18 publications for review” and yet elsewhere I quote “After the initial candidates were selected, they were subjected to eliminating evaluations”. I do not think the term “candidate” can be used to refer to records retrieved in reviews. You may want to rephrase those and elsewhere (Introduction and Methods sections) in the body of your text and use “records” or “articles” instead.

Authors' response: The word “candidate” has been rephrased to “article” throughout the manuscript.

3. Your “results” and “conclusions” subsections of the Abstract are not robust in a way that helps the reader understand what you found and what you learned or deduced from the findings and your recommendations. Kindly include a sentence or two each for personal protective equipment, testing methods, medical treatment, and other considerations in the “results” subsection.

Authors' response: A summary sentence has been added into each subsection of “results” so that “results” can be seen as more separate from “conclusion.”

4. Kindly include this phrase in the “methods” subsection of your Abstract: “The keywords ‘COVID-19 technology,’ ‘COVID-19 invention,’ and ‘COVID-19 equipment’ were used in a Google search to generate related news articles and scientific reports.” Moreover, indicate the date that the search was performed.

Authors' response: The sentence has been added into the abstract.

5. Regarding your PRISMA diagram, your numbers for records identified from other databases (websites) do not add up. You excluded 15 articles from 30 you sought to retrieve, and it follows that you apparently excluded all 15 articles you assessed for eligibility, but you contradictorily still included the 15 articles in this review. Kindly verify and correct your PRISMA chart.

Authors' response: The PRISMA chart has been updated.

6. Your PRISMA diagram shows that you searched other websites other than Google, it will be absolutely helpful and more robust to indicate these websites under your “Search strategy.”

Authors' response: Another website has been included in the search strategy.

7. You did well to have included the PRISMA flow. Kindly substantiate your phrase I quote “The selection of the article followed the guideline of PRISMA 2020” with a suitable reference.

Authors' response: Two new references have been added to the manuscript.

8. Under “Testing methods” in your Results section, kindly also allude to “pooled” and “rapid testing (serology and antigen)” technologies as these are indispensable innovations to increasing the turnaround time and for timely detection. This updated Cochrane review as well as this list of 42 rapid testing technologies considered to be of acceptable performance by the UK government can help you identify suitable new technologies to add to this review. Regarding their pros and cons, it might be worthwhile to also look at the extent to which information provided by manufacturers is helpful for each technology considered if possible.

Authors' response: The section of “testing methods” has been edited, and more references including have been added to Table 2 of the manuscript. Additionally, an additional paragraph has been added to the manuscript.

9. Coming to your Study Limitations, your phrase, and I quote “Also, the paper only provides a quantitative comparison between the technologies” does not seem to be coherent with your synthesis approach. I think this should be a qualitative comparison since you made use of textual descriptions to draw similarities and dissimilarities between the data. Tabular presentations facilitate the narrative but do not make it quantitative. Kindly phrase and include the following in your Study Limitations as well;

a. The search strategy was not comprehensive as it was limited to one database (Google).

b. The fact that the protocol was not registered with PROSPERO might have affected the results in one way or the other.

c. Even though you unveiled some of the complexities regarding supporting technologies, a quantitative analysis would have also added value to the review results.

d. You did not do a formal appraisal of the included studies and the overall evidence from included studies. This must-have affected your results.

10. Your Table 1 through 3 make up 17 articles instead of 18 according to the number of retained articles. Kindly verify.

Authors' response: The study limitation has been edited. There are 23 articles in the manuscript.

##### Minor Comments

11. Your “Conflicts of Interest” should follow the journal guidelines. Kindly use “None declared.”

Authors' response: The statement has been updated.

## Round 3 Review

### Reviewer CM [3]

Authors' response: We would like to express our gratitude again to the reviewers for their careful thoughts. We have carefully considered, responded to, and made changes to the manuscript based on the specific recommendations of the reviewers. We are very appreciative of the kind comments. Thank you.

Our point-by-point responses to the comments of the reviewers are listed as follows:

#### General Comments

Unfortunately, I still have the 3 following concerns.

#### Specific Comments

##### Major Comments

1. Recommendation #3: I am happy that the authors of this paper [3] improved on the results following the recommendation in Point 3, but this recommendation was primarily referring to the Results and Conclusion subsections in the Abstract. The current wordings in the Results of the Abstract should be moved to the Methods subsection of the Abstract. This means that you are yet to produce a summary of your findings (results) in the Abstract. Moreover, kindly word up the Conclusion subsection in the Abstract to reflect the main Conclusion of the paper.

Authors' response: The abstract section has been updated.

2. The authors have also done well to have deployed the current PRISMA flow chart. However, your flow diagram shows that you included 5 articles from a previous version of this review indicating this paper is about updating a previous review and I do not think it is the case [1]. Except otherwise, kindly leave this box empty and move this number (n=5) to either “Records identified from Databases” or “Records identified from Websites”. My humble suggestion is that since you seem to have identified 200 records from Google search and ScienceDirect, under “Records identified from Databases (n=200)”, kindly specify “Google=150”, and “ScienceDirect=50” for readers to be clear about how many articles were retrieved from which database. Under “Records identified from Websites, kindly put “n=5, assuming that the 5 previously published reviews were identified from websites. If these were identified through Google search, ScienceDirect, or Cochrane, then kindly include under Records identified from Databases and leave “Records identified from Websites empty.

Authors' response: The flowchart has been improved according to the suggestions. The five review articles have been identified from Database, so the boxes of “identification of new studies via other methods” as well as “previous studies” has been removed according to the study by Page et al, “PRISMA 2020 explanation and elaboration: updated guidance and exemplars for reporting systematic reviews.” Please kindly see the manuscript.

3. You need to correct your statement “Three previous review papers were also included” as this seems to be 5 in the flow diagram.

Authors' response: The statement has been corrected.
